# Structural Fire Performance of Concrete-Filled Built-Up Cold-Formed Steel Columns

**DOI:** 10.3390/ma15062159

**Published:** 2022-03-15

**Authors:** Hélder D. Craveiro, Rohola Rahnavard, José Henriques, Rui A. Simões

**Affiliations:** 1 Department of Civil Engineering, ISISE, University of Coimbra, Rua Luís Reis Santos, 3030-790 Coimbra, Portugal; rahnavard@uc.pt (R.R.); rads@dec.uc.pt (R.A.S.); 2 Faculty of Engineering Technology, CERG, University of Hasselt, BE 3590 Diepenbeek, Belgium; jose.gouveiahenriques@uhasselt.be

**Keywords:** cold-formed steel, built-up section, concrete-filled, load-bearing capacity, local-global buckling interaction, global buckling, fastener spacing

## Abstract

Concrete-filled composite columns are widely used in the construction industry, exploiting the benefits of combining steel and concrete, providing, for instance, high load-bearing capacities and enhanced fire resistance. These solutions are extensively used in high-rise buildings and/or when high fire resistance performance requirements are imposed. In this exploratory research, a new type of concrete-filled composite column is investigated using fire resistance tests. Promoting the use of cold-formed steel products and developing innovative solutions for low-rise buildings with lower passive fire protection requirements led to the solutions presented in this research. Hence, a set of fire-resistance tests were undertaken on concrete-filled closed built-up cold-formed steel columns, where single cold-formed steel shapes are combined and fastened to create a box-shaped cross-section. The experimental results were then compared with the corresponding bare steel solutions to assess, in detail, the observed enhancements. Additionally, the effect of restraint on thermal elongation was assessed.

## 1. Introduction

Concrete-filled composite columns are well known for their high load-bearing capacity, increased stiffness, high ductility, energy absorption, and enhanced structural fire performance [1,2]. These solutions are commonly used in high-rise buildings and have been extensively investigated at ambient temperature and fire conditions [2,3,4,5,6]. The most common configurations, using circular, square, rectangular, and elliptical steel tubes, have been investigated [2], and new shapes and solutions are being explored, for instance, including additional inner steel profiles (tubes or hot-rolled H sections), creating the so-called concrete-filled double-skin composite columns [7,8,9,10], and concrete-filled composite columns with embedded steel profiles [11,12,13].

Concrete-filled tubular solutions are predominantly being used in high-rise buildings where high load-bearing capacity and fire resistance is required. However, for low- and mid-rise buildings, the use of composite concrete-filled members is less common. This is mainly due to the lack of versatility of existing solutions and the consideration of prefabrication and modularity as specific requirements to tackle. There is an urge in the construction sector to develop and promote prefabricated solutions with a higher level of versatility. Cold-formed steel (CFS) products present themselves as highly versatile solutions that may be widely used in low- to medium-rise buildings, with significant environmental benefits. Moreover, optimal solutions can be specified for a wider range of scenarios when compared with the more traditional concrete-filled solutions using standard hollow steel sections. Despite the advantages of cold-formed products, such as versatility, lightness, high strength-to-weight ratio, ease of erection and transportation [14,15], one of the weakest points concerns reduced structural fire resistance due to reduced thickness, a high section factor and the high thermal conductivity of steel. Consequently, the use of passive fire protection in cold-formed steel structures is required. To further expand the applicability of CFS products in the construction sector, it is possible to combine multiple individual section shapes to fabricate built-up tubular members, which can span a greater distance, have a higher load-bearing capacity, and have greater torsional stiffness. Additionally, built-up elements are often symmetric, avoiding eccentricities between shear and gravity centres, contributing to higher member stability.

Additionally, the fabricated closed built-up CFS sections can be easily filled with concrete to address existing shortcomings related to the low load-bearing capacity of individual members, low fire resistance, and plate stability due to the reduced thickness of the individual members. The concrete infill will substantially increase the load-bearing capacity of CFS columns and their fire resistance while mitigating local buckling phenomena due to the confinement provided by the concrete which prevents inward deformation of the plate elements [16]. This phenomenon can effectively contribute to fully exploring the mechanical properties of steel, promoting the use of high-strength steel. This new solution can also be considered to compete with light-steel framing solutions, with enhanced structural performance and fire resistance.

The number of hybrid or multi-material structural solutions in use will increase significantly, targeting enhanced structural performance [17]. The composite behavior of CFS-concrete members has been investigated in the past, where the CFS profiles have also been considered as a replacement for traditional reinforcement bars, and others assessing the benefits and potential of composite structures combining CFS products and concrete [18,19,20,21,22,23,24,25,26,27,28,29,30,31]. Several studies were undertaken to investigate the behavior of shear connectors and composite beams [19,20,22,27,28,29,30,31], showing high levels of ductility and load-bearing capacity. Other composite solutions incorporating CFS and concrete have been recently presented, further exploring the versatility of CFS products [32,33,34]. However, the proposed solutions are mainly used in composite beams, with few contributions or proposals for composite solutions for columns for low- to mid-rise buildings.

In this exploratory research, this synergy between CFS and concrete is investigated under fire conditions with restrained thermal elongation (considering indirect fire actions), assessing in detail the behavior of this new type of concrete-filled closed built-up CFS composite columns.

## 2. Materials and Methods

### 2.1. Test Specimens and Plan

**Figure 1:**
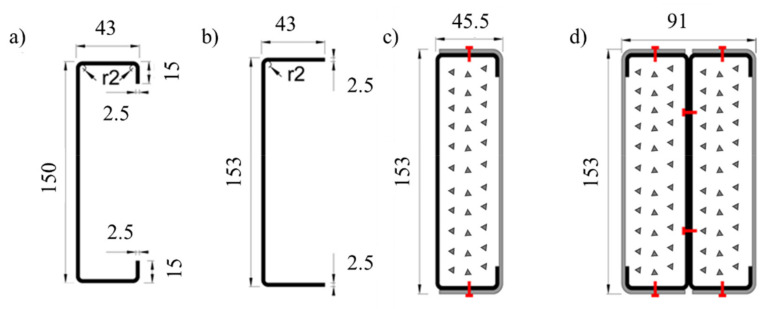
(**a**,**b**) Dimensions of the individual cross-sections. (**c**,**d**) Tested composite cross-section shapes. (**c**) Composite R-section. (**d**) Composite 2R-section.

This research investigated the behavior of concrete-filled closed built-up cold-formed steel composite columns using experimental tests. Two basic configurations were selected, comprising two commercially available cold-formed steel sections, namely, lipped channels (C150 × 43 with t = 1.5 mm—Figure 1a) and plain channels (U153 × 43 with t = 1.5 mm—Figure 1). The first cross-section consisted of a lipped channel and a plain channel, positioned in such a way to obtain a box shape (R cross-section—Figure 1c). The plain channel and the lipped channel were fastened in the flanges using self-drilling screws along the length of the column. Finally, the 2R cross-section consisted of two-lipped channels (C) positioned back-to-back (I) and fastened in the web, and two plain channels (U) positioned in such a way to obtain a box shape (Figure 1d). The length of all profiles was 2950 mm, and the fasteners’ spacing along the column’s length was 725 mm. The spacing adopted for the self-drilling screws was based on the observation of designed CFS structures and preliminary numerical simulations, aiming to ensure reasonable levels of composite action between the individual shapes. It is worth mentioning that the selected distance between fasteners would still allow for the occurrence of local buckling phenomena. The individual shapes were fastened together using Hilti S-6.3×1.9MD03Z self-drilling screws (Hilti, Portugal).

All individual profiles were manufactured with S280GD+Z steel; they were then hot-dip galvanized with zinc on each side (PERFISA S.A., Portugal). According to EN 10147, the yield strength is 280 MPa, and the ultimate strength is 360 MPa.

The closed built-up cold-formed steel columns were filled with C20/25 normal-weight concrete, aiming to accurately assess the impact of the concrete infill on the overall closed built-up CFS columns in terms of structural fire performance mitigating local buckling phenomena. In this investigation, eight fire-resistance tests were conducted, considering the influence of restraint on thermal elongation, hence assessing in detail the influence of indirect fire actions. Additionally, both pinned and fixed boundary conditions were considered. There were two repetitions per tested configuration and boundary condition.

In the fire resistance tests of the concrete-filled composite columns, the concrete infill was considered as a fire protection material (passive fire protection material); hence, the adopted service load in the experimental tests was based on design buckling load of the bare steel built-up CFS columns determined according to the EN 1993-1-1 [35] and EN 1993-1-3 [36]. In this study, the adopted service load corresponded to 50% of the design buckling load, $N_{b,Rd}$. The EN 1993-1-3 [36] adopts the Effective Width Method (EWM), and the design buckling load is determined according to the following equations:

(1) $$N_{b,Rd}=\frac{\chi A_{eff}f_{y}}{\gamma_{M1}}$$

(2) $$A_{eff}=t\left[h_{e1}+h_{e2}+2b_{e1}+2\left(b_{e2}+c_{e2}\right)\chi_{d}\right]$$

(3) $$b_{eff},\;c_{eff}=\rho \;.\;b$$

(4) $$\sigma_{cr}=\frac{k_{\sigma }\pi^{2}E}{12\left(1-\upsilon^{2}\right){\left(b/t\right)}^{2}}$$

(5) $$\sigma_{cr,s}=\frac{2\sqrt{KEI_{s}}}{A_{s}}$$

(6) $$\chi =1/\left(\Phi +\sqrt{\Phi^{2}-{\bar{\lambda }}^{\;2}}\right)$$

(7) $$\Phi =0.5\left[1+\alpha \cdot \left(\bar{\lambda }-0.2\right)+{\bar{\lambda }}^{\;2}\right]$$

(8) $$\bar{\lambda }=\sqrt{\frac{A_{eff}f_{y}}{N_{cr}}};\;N_{cr}=\min\left\{N_{cr,F},\;N_{cr,T},\;N_{cr,FT}\right\}$$

(9) $$N_{cr,F}=\frac{\pi^{\;2}\;E\;I}{L_{e}^{2}}=\frac{\pi^{2}\;E\;A}{(L_{e}/r{)}^{2}}$$

where:
$N_{b,Rd}$Design buckling resistance of a compression memberχReduction factor for relevant buckling mode$A_{eff}$Effective are of a cross-section$f_{y}$Yield strength$\gamma_{M1}$Partial safety factor for resistance of memberstPlate thickness$h_{ei}$Effective height of a cross-section$b_{ei}$, $b_{eff}$Effective width of a cross-section, effective plate width$c_{ei}$Effective lip length of a cross-section$\chi_{d}$Reduction factor for the distortional buckling resistanceρReduction factor for plate bucklingbWidth of a cross-section, plate width$\sigma_{cr}$Elastic critical plate buckling stress$k_{\sigma }$Buckling factor depending on the stress distribution and boundary conditions of the plateEModulus of elasticityυPoisson’s ratio $\sigma_{cr,s}$Elastic critical buckling stress for an edge stiffenerKSpring stiffness per unit length of a stiffener$I_{s}$Effective second moment of area of the stiffener$A_{s}$Effective area of a stiffener${\bar{\lambda }}^{\;}$Relative slenderness of a memberαImperfection factor$N_{cr,F}$Elastic flexural buckling force$N_{cr,T}$Elastic torsional buckling force$\;N_{cr,FT}$Elastic torsional-flexural buckling force$L_{e}$Effective length of a memberrRadius of hydration of a cross-section

In Table 1 the predicted values for the design buckling loads for the different cross-sections are presented.

### 2.2. Test Set-Up and Procedure

**Figure 2:**
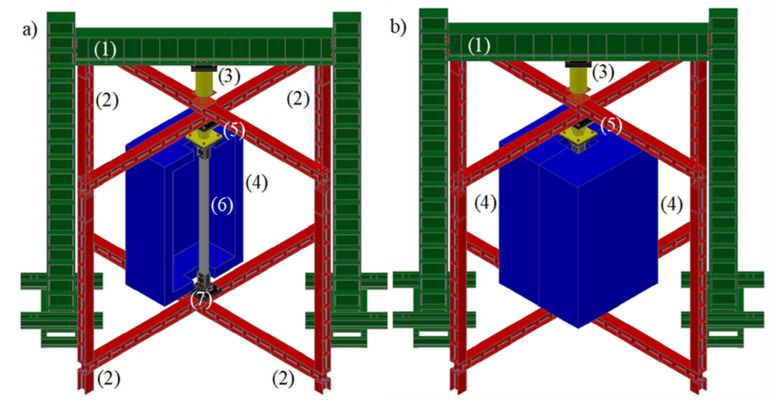
Schematic view of the experimental test set-up. (**a**) 3D view with the furnace open. (**b**) Furnace closed.

**Figure 3:**
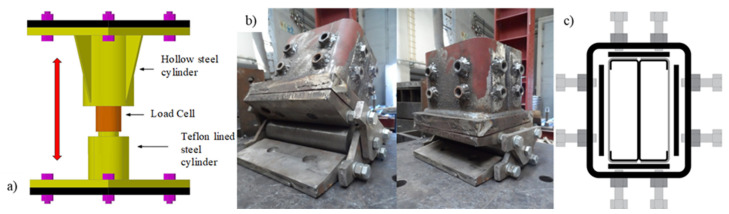
(**a**) Schematic view of the device built to measure the generated axial forces. (**b**) Fabricated support device to reproduce the boundary conditions in the tests and (**c**) adjustment mechanism to fix the specimens to the support device.

The fire resistance tests were conducted considering the influence of indirect fire actions, hence considering the influence of restraint on thermal elongation. As previously mentioned, the service load applied to the columns corresponded to 50% of the design buckling load. Consequently, the columns initially loaded up the desired service load, using force control, which was kept constant during the fire resistance test. In Figure 2, a schematic global view of the test set-up is presented. The test set-up comprised two large steel frames, one supporting the loading mechanism ((1)—reaction frame) and the other supporting the boundary conditions and specimen and providing the required restraint to thermal elongation to the column in fire ((2)—restraining frame). The imposed axial restraint of the concrete-filled built-up cold-formed steel column was approximately 3 kN/mm. The axial restraint to thermal elongation imposed to the composite column was attained by blocking the vertical rigid body movement of the top orthogonal beams at the supports. A hydraulic jack (3) with a maximum load capacity of 3000 kN and a maximum stroke length of 300 mm was fixed to the reaction frame. A servo-hydraulic central unit controlled the hydraulic jack. The thermal action was applied by a vertical modular electric furnace ((4) in Figure 2) programmed to reproduce the standard fire curve ISO 834. This electric furnace was composed of three vertical modules that could open in a split system. Two of the vertical modules were 1 m (90 kVA each module) and the last one 0.5 m height (45 kVA). When the two parts of the furnace were closed, a chamber (2.5 m × 1.5 m × 1.5 m) was created around the test column [37]. To measure the indirect actions generated during the heating phase of the fire resistance test, a specific device was built (5) (Figure 3a).

The device depicted in Figure 3a (steel piston device with interior load cell) measured the additional axial forces generated during the heating phase. The device consists of a hollow steel cylinder where a stiff solid steel cylinder Teflon lined (PTFE) slides through it. A 500 kN load cell was positioned on the top of the stiff steel cylinder. During the fire test, the load cell was compressed against the top end-plate of the hollow steel cylinder, measuring the additional axial forces generated due to the imposed restraint to thermal elongation.

To fix the columns, a special device was also developed to simulate adequately the desired boundary conditions (Figure 3b). The developed system allowed one to fit different cross-section shapes (Figure 3c). Moreover, at both ends, a rigid end-plate was positioned, ensuring that shear and warping deformations at the boundary conditions were restrained.

**Figure 4:**
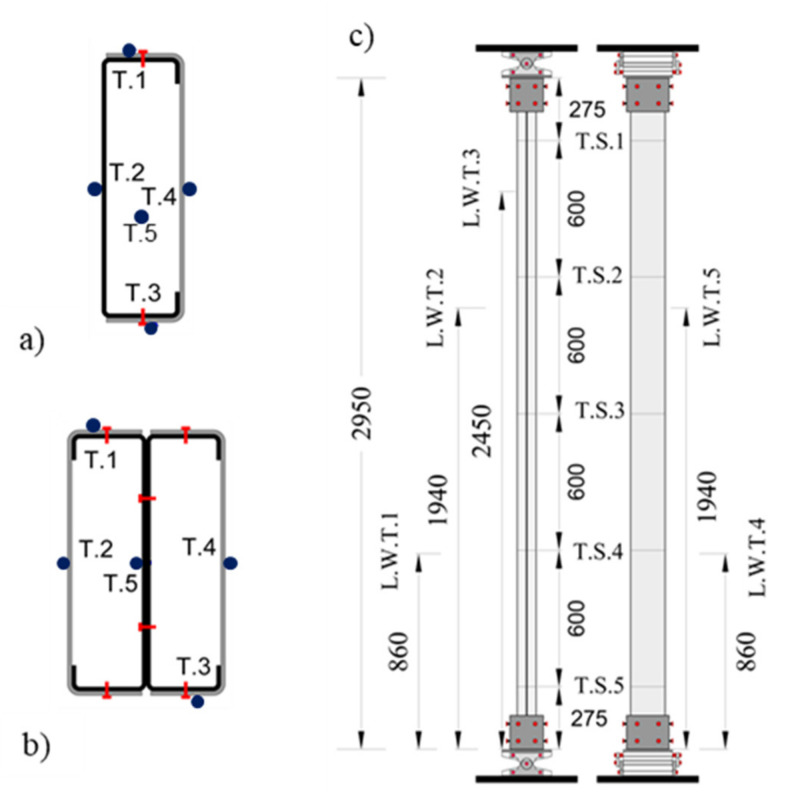
Instrumentation used and corresponding location (all dimensions in mm). (**a**,**b**) Position of thermocouples. (**c**) Position of thermocouples and transducers along the length of the column.

All specimens were instrumented with thermocouples in different positions at the cross-section level and in different locations along the length of the column. Additionally, the service load generated axial forces, and vertical and lateral displacements were monitored during the tests. In Figure 4, the instrumentation used, and its location, is depicted at the cross-section level and along the length of the column.

The fire resistance test with restrained thermal elongation consisted of several steps:

- Applying the service load (50% $N_{b,Rd}$) under force-control.
- After reaching the desired service load, the rigid-body movement of the top beams of the surrounding steel frame was blocked, ensuring that the axial restraint to thermal elongation was acting before turning on the electrical furnaces. For these specific tests, the imposed restraint to the composite column in fire was 3 kN/mm.
- Turning on the electrical furnaces programmed to follow as closely as possible the ISO 834 fire curve.
- With the temperature increase, the composite column started to expand. However, the column could not expand freely due to the imposed restraint. Consequently, additional axial forces were generated (indirect fire actions). The combination of additional axial forces and degradation of mechanical properties of steel and concrete led to the collapse of the composite columns. When the axial forces started to decrease and once again reached the initial service load, the composite column was no longer able to withstand the initially applied service load. This time instant defines the fire resistance of the column in terms of load-bearing capacity.

## 3. Experimental Results and Discussion

### 3.1. Material Characterization

**Figure 5:**
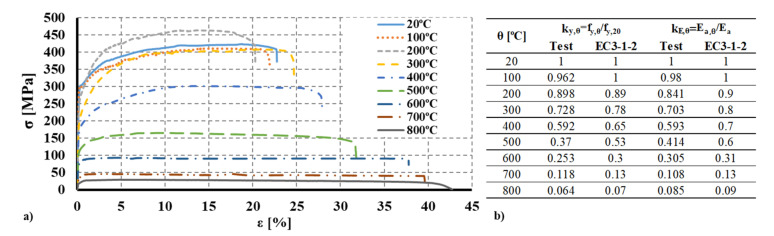
(**a**) Stress-strain curves at elevated temperature [38]. (**b**) Reduction factors for the mechanical properties from the tests and EN 1993-1-2.

In the scope of this investigation, the mechanical properties of CFS were also determined at ambient temperature. In a previous study, the mechanical properties of the S280GD+Z steel were assessed [38]. Aiming to accurately determine the degradation of the mechanical properties as a function of temperature, tensile tests were conducted according to the ISO 6892-1 [39] for temperatures ranging from 20 °C to 800 °C considering 100 °C steps. Steady-state tests were conducted, hence heating the coupon specimens to the established temperature and then performing the tensile test. Considering that the same steel grade was used and that the supplier was the same, similar degradation of the mechanical properties may be assumed. In Figure 5, the obtained stress-strain curves and specimens after the test are presented for the S280GD+Z steel. Based on the experimental data, it was possible to determine the reduction factors for the yield strength and modulus of elasticity with increasing temperature (Figure 5b)). Comparing the results with the data provided by the EN 1993-1-2 [40] (clause 3.2, Table 3.1 and Annex E, Table E.1), it was observed that the degradation of the mechanical properties was higher for all tested temperatures.

Additionally, it was observed that at 200 °C the ultimate strength ($f_{u,200\;{}^{\circ}C}$) was higher than at ambient temperature and 100 °C. This singularity was observed and reported by several authors for low-strength cold-formed steels [41,42] and may be related to the chemical composition of the steel. Chemical reactions and transformations may occur in the steel base, leading to an increase in the ultimate strength. For higher temperatures, this singularity was no longer observed.

### 3.2. Failure Modes

**Figure 6:**
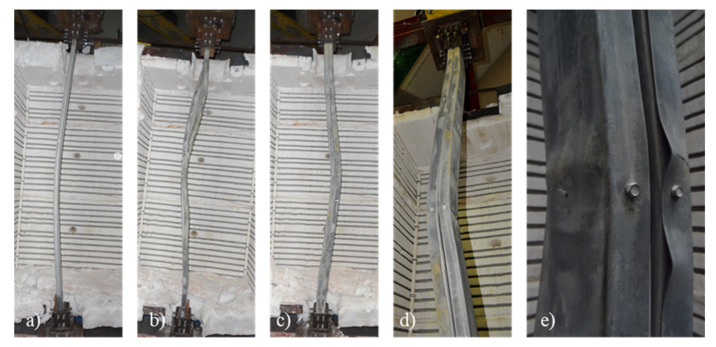
Observed failure modes. (**a**) R composite column with pinned boundary condition. (**b**) R composite column with fixed boundary condition. (**c**–**e**) 2R composite column with fixed boundary condition.

In Figure 6a,b, the final deformed shapes are presented for some of the specimens and both pinned and fixed boundary conditions. For columns with closed built-up R cross-section, the predominant failure mode was the interaction between flexural buckling aboutthe minor axis and local buckling around mid-height of the column for both end-support conditions. For the semi-rigid end-support condition, local buckling was also observed at 50 cm of the end-support devices. Points of inflection in the final deformed shape for the semi-rigid end-support condition are recognizable.

For columns with closed built-up 2R cross-section (Figure 6c–e), the predominant failure mode was the interaction between flexural buckling around the minor axis and distortional and local buckling at around mid-height of the column. For the 2R cross-section, local buckling is recognizable. It is worth mentioning that distortional and local buckling play a more relevant role in the failure of columns with 2R cross-section. Distortional buckling was observed for the external plain channels (U-shape profiles) immediately after the location of the self-drilling screws (Figure 6e). Once again, for the semi-rigid end-support condition and the 2R cross-section, distortional buckling was observed near the end support devices. Moreover, the points of inflection in the final deformed shape are recognizable.

It is worth mentioning that the inward movement of the plates of the individual plates was restrained by the presence of the lightweight concrete infill, which contributes to minimizing the susceptibility of such members to local buckling phenomena. This is an important advantage since the mechanical characteristics of the steel can be further exploited.

### 3.3. Temperature and Fire Resistance Time

In this study, the investigated structural members’ fire resistance (load-bearing capacity) was assessed in terms of time and temperature. From the experimental results, it was possible to extract the most relevant results in terms of critical temperature (temperature from which the column was no longer able to withstand the applied service load), the critical time, the maximum axial load ($P_{max}$), and the corresponding temperature and finally the ratio ($P_{max}/P_{0}$) between the maximum axial load recorded ($P_{0}$ plus the generated axial forces, P) during the test due to the additional axial forces generated due to the influence of restraint to thermal elongation ($P_{max}$) and initial service load ($P_{0}$). Along the length of the column, a relevant thermal gradient was observed, with lower temperatures recorded by the thermocouples located at both ends of the columns (T.S.1 and T.S.5). Hence, the peak temperature corresponding to the maximum axial load and the critical temperature was determined considering the average values recorded by the thermocouples in T.S.2, T.S.3, and T.S.4. The main results are presented in Table 2, Table 3, Table 4, and Table 5, respectively, for pinned R columns, fixed R columns, pinned 2R columns, and finally fixed 2R columns. The fire resistance in the time domain is represented by the critical time ($t_{cr}$), whereas the temperature domain is represented by the critical temperature ($\theta_{cr}$). The critical temperature ($\theta_{cr}$) defines the temperature from which the column is no longer able to withstand the applied service load and fails due to the combined effect of the applied load and degradation of the mechanical properties with increasing temperatures. For the concrete-filled closed built-up R specimens, the fire resistance time ranged from 25.1 to 29 min, whereas for the doubly symmetric concrete-filled closed built-up 2R specimens, the fire resistance time was higher than 30 min. Additionally, the maximum axial compression load and the maximum value of the ratio $P/P_{0}$ are presented in Table 2, Table 3, Table 4 and Table 5.

**Figure 7:**
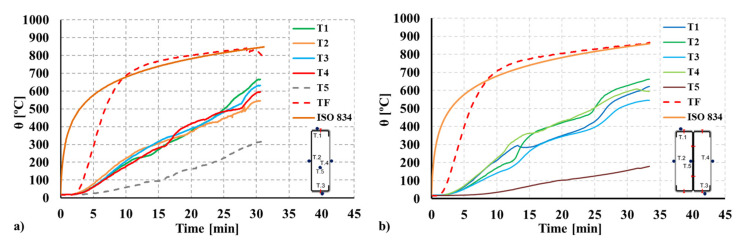
Temperature evolution monitored during the tests for the T.S.3 section. (**a**) R composite section. (**b**) 2R composite section.

The temperature evolution at the cross-section level was assessed for both configurations tested and the temperature in the chamber of the furnace. For both R and 2R composite sections, the monitored temperature at every location specified in Figure 4 and at mid-height of the column (T.S.3) is plotted in Figure 7. Additionally, thermocouples were positioned in the concrete (R) and the internal steel plates fully embedded in concrete (2R). For the doubly symmetric 2R section, the inner plate temperature is below 200 ºC during the test.

As expected, the highest critical temperature was obtained for the doubly symmetric concrete-filled closed built-up 2R CFS columns of around 527 ºC. In terms of the $P/P_{0}$ ratio, the maximum value (1.61) was obtained for the mono-symmetric closed built-up R CFS.

### 3.4. Evolution of Restraining Forces and Fire Resistance

**Figure 8:**
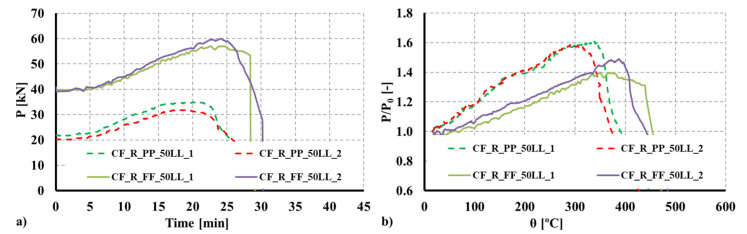
(**a**) Evolution of axial forces due to restraint to thermal elongation for the composite R section. (**b**) Evolution of the P/P_0_ ratio for the composite R section.

**Figure 9:**
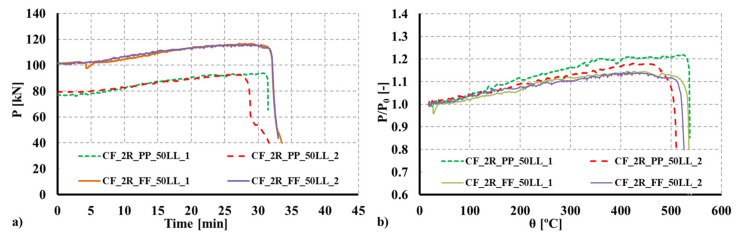
(**a**) Evolution of axial forces due to restraint to thermal elongation for the composite 2R section. (**b**) Evolution of the P/P_0_ ratio for the composite 2R section.

Since restraint to thermal elongation was a specific parameter investigated in the experimental tests, additional axial forces generated during the fire test were monitored. In Figure 8 and Figure 9, the obtained results are plotted both in terms of the actual forces generated and the non-dimensional ratio $P/P_{0}$ (P—generated axial forces; $P_{0}$—initial service load) as a function of time and the average temperature of the column. As previously mentioned, during heating, additional axial forces were generated due to thermal elongation and the imposed restraint provided by the surrounding structure. Simultaneously, and with temperature increase, the mechanical properties of steel and concrete degraded up to a point where the column could no longer withstand the applied service load and the additional forces generated.

Analyzing the experimental results, it was observed that the ratio $P/P_{0}$ increased up to 1.595 and 1.443 for the R columns, respectively, for the fixed and pinned boundary conditions, whereas for the 2R columns the ratio increased up to 1.14 and 1.20, respectively, for the fixed and pinned boundary conditions. Comparing the obtained ratios as a function of the adopted boundary conditions, it was observed that for the R columns the ratio $P/P_{0}$ was 8.04% higher for pinned columns, whereas for the 2R columns, the ratio $P/P_{0}$ was 5.3% higher for pinned columns. All curves plotted in Figure 8 and Figure 9 show a sudden drop after $P_{max}$ is reached.

It is worth mentioning that considering the influence of restraint on thermal elongation, the failure time and temperature will be lower than for the scenarios where axial restraint to thermal elongation is not considered. According to the EN 1993-1-2 [40], conducting member analysis, the effect of axial or in-plane thermal expansions may be neglected. The obtained results show that the restraints imposed by the surrounding structure to individual members subjected to fire may significantly influence their structural fire behavior.

## 4. Comparison with Bare Steel CFS Built-Up Columns

**Figure 10:**
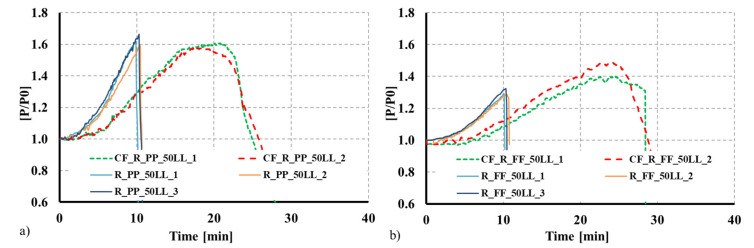
Comparison between bare steel CFS 2R columns and concrete-filled CFS 2R columns as a function of time. (**a**) Pinned columns. (**b**) Fixed columns.

**Figure 11:**
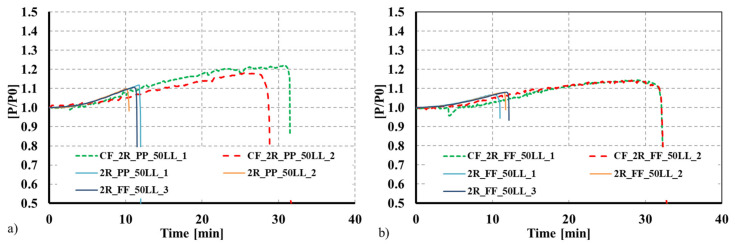
Comparison between bare steel R columns and concrete-filled CFS R columns as a function of time. (**a**) Pinned columns. (**b**) Fixed columns.

To assess the proposed solution’s validity and efficiency, the observed results were compared with results for similar solutions but without the concrete infill [43,44], and a different plate thickness for the individual CFS profiles. The adopted thickness was 1.5 mm for the composite sections, whereas for the bare steel built-up solutions, the adopted thickness was 2.5 mm. As can be observed in Figure 10 and Figure 11, even though the thickness of the steel profiles used in the composite columns was 1.5 mm, that the fire resistance time greatly increased when the concrete infill was used. For instance, for the composite 2R columns, the fire resistance time was 33 min, representing a 153.8% increase compared with the 2R bare CFS column with a plate thickness of 2.5 mm. The concrete infill significantly enhances the overall fire performance of the CFS built-up columns. Moreover, the concrete infill can greatly contribute to mitigating the susceptibility of the thin-walled CFS profiles to local buckling phenomena, hence exploiting the full resistance of the employed steel. This can even promote the use of high-strength steel for composite applications.

This solution will open new market opportunities for CFS products, allowing the development of new structural systems or strengthening solutions for existing ones. Such a type of composite solution considering steel as the main material also presents the potential for prefabrication and modularity, and moving construction operations to the workshop, creating, for instance, new market opportunities for prefabricated steel-concrete composite solutions. Future research should consider the use of lightweight materials as infill, such as lightweight or ultralightweight concrete.

## 5. Conclusions

The benefits of the synergies created between CFS and concrete are clear from the experimental tests, where these new solutions presented an improved structural fire performance, with an increase of 153% in terms of fire resistance for some of the established comparisons. Additionlly, the impact of restraint on thermal elongation was assessed. During heating and due to the imposed restraint, additional axial forces are generated, hence reducing the fire resistance and, consequently, the critical temperature. It is also worth noting the advantages of using such a composite solution to mitigate the local buckling phenomena of the thin-walled steel plate elements.

Finally, the observed results show that composite CFS-concrete solutions should be further explored and investigated, taking advantage of the versatility of CFS products. Such versatility enables the adoption of optimal solutions contributing to the reduce the consumption of raw materials in the construction sector. Moreover, it is envisaged that such solutions have the potential to create a new structural solution for low- to mid-rise buildings, targeting prefabrication, industrialization, and modularity, with reuse in mind.

## Figures and Tables

**Table 1:** Predicted values for CFS columns ($N_{b,Rd}$) and applied service loads ($P_{0}$).

	R		2R	
	t = 1.5 mm		t = 1.5 mm	
	Pinned	Fixed	Pinned	Fixed
*N_b_*_,*rd*_ (kN)	45.35	81.58	159.06	203.82
*P*_0_ (kN)	22.68	40.79	79.53	101.91

**Table 2:** Experimental results for pinned R columns.

Test Reference	*θ_peak_* (°C)	*P_max_* (kN)	*P_max_/P*_0_ (––)	*θ_cr_* (°C)	*t_cr_* (min)
R_PP_50LL_K1-1	339	35	1.61	392	25.1
R_PP_50LL_K1-2	290	32	1.58	374	26
μ	314.4	33.5	1.595	383.2	25.458
σ	24.477	1.500	0.011	8.860	0.392

**Table 3:** Experimental results for fixed R columns.

Test Reference	*θ_peak_* (°C)	*P_max_* (kN)	*P_max_/P*_0_ (––)	*θ_cr_* (°C)	*t_cr_* (min)
R_FF_50LL_K1-1	369	57	1.40	442	28.5
R_FF_50LL_K1-2	388	60	1.49	443	29
μ	379.6	58.5	1.443	442.4	28.667
σ	9.237	1.500	0.046	0.541	0.167

**Table 4:** Experimental results for pinned 2R columns.

Test Reference	*θ_peak_* (°C)	*P_max_* (kN)	*P_max_/P*_0_ (––)	*θ_cr_* (°C)	*t_cr_* (min)
2R_PP_50LL_K1-1	487	94	1.22	537	31.5
2R_PP_50LL_K1-2	484	93	1.18	507	29
μ	485.6	93.4	1.2	521.6	30.1
σ	1.112	0.400	0.001	15.036	1.392

**Table 5:** Experimental results for fixed 2R columns.

Test Reference	*θ_peak_* (°C)	*P_max_* (kN)	*P_max_/P*_0_ (––)	*θ_cr_* (°C)	*t_cr_* (min)
2R_FF_50LL_K1-1	482	117	1.15	534	32.3
2R_FF_50LL_K1-2	451	116	1.14	521	32.1
μ	466.4	116.3	1.14	527.4	32.2
σ	15.194	0.300	0.002	6.263	0.017

## Data Availability

The data presented in this study are available on request from the corresponding author.
